# Enhancing analgesia in acute renal colic pain: a randomized controlled trial of gabapentin adjunct to ketorolac-based regimen

**DOI:** 10.3389/fpain.2024.1427711

**Published:** 2024-10-14

**Authors:** Parisa Kianpour, Parmis Valavioun, Pooya Payandemehr, Arash Safaei, Yasaman Borhani, Hooshyar Honarmand, Mojtaba Mojtahedzadeh, Kamal Basiri, Elahe karimpour-Razkenari, Farhad Najmeddin

**Affiliations:** ^1^ Anesthesia, Critical Care and Pain Management Research Center, Tehran University of Medical Sciences, Tehran, Iran; ^2^ School of Pharmacy, Tehran University of Medical Sciences, Tehran, Iran; ^3^ Department of Emergency Medicine, Sina Hospital, Tehran University of Medical Sciences, Tehran, Iran; ^4^ Department of Clinical Pharmacy, School of Pharmacy, Tehran University of Medical Sciences, Tehran, Iran

**Keywords:** renal colic pain, gabapentin, ketorolac, morphine, somatic pain

## Abstract

**Background:**

Renal colic is characterized by severe pain that is highly disabling. Gabapentin, an antiepileptic medication, is often recommended as a first-line therapy for neuropathic pain. However, its effectiveness in managing somatic pain, which is defined as the result of activity by pain receptors in the deep tissues, such as renal colic pain, is not as well-established.

**Method:**

A phase 3 randomized clinical trial was conducted to evaluate the adjuvant analgesic effects of gabapentin on acute renal colic pain. Eligible patients participated in the study via random allocation to the control or gabapentin groups using the block randomization method. All patients received a shared regimen of ketorolac and rescue morphine as the conventional analgesic treatment for renal colic pain. Gabapentin was added as an adjuvant analgesic for the gabapentin group.

**Result:**

A total of 63 individuals with an average age of 41.35 ± 13.08, were enrolled and completed the study. At the time of admission, there were no significant differences between the baseline characteristics of two groups, with exception of weight. The gabapentin group showed a significantly higher percentage of patients with pain severity of less than 5 after 60 and 90 min, as well as a significantly lower percentage of morphine rescue requirement and total morphine intake (mg) and mg/kg.

**Conclusion:**

In cases of acute renal colic, gabapentin significantly decreases both the amount of morphine required and the degree of pain, indicating that it may be a useful adjutant to standard analgesic regimens. Treatment regimens that include gabapentin may help individuals manage their pain and become less reliant on opioids.

**Clinical Trial Registration:**

https://irct.behdasht.gov.ir/trial/56066, identifier: IRCT20200322046833N2.

## 1 Introduction

Renal colic pain is an acute and catastrophic pain caused by kidney stones, affecting 5%–15% of people worldwide (1, 2). Treatment aims to lessen the patient's discomfort and preserve renal function by exerting the stone (1). The effective and safe management of renal colic pain remains a therapeutic challenge in the emergency department.

Since increased ureteral smooth muscle contractile activity is the primary cause of renal colic pain, smooth muscle relaxants are helpful for managing pain and facilitating the elimination of stones (1, 3, 4).

Currently, a range of medications can be used for the management of renal colic, such as calcium channel blockers, alpha receptor antagonists, and antimuscarinic medicines. However, opioids and nonsteroidal anti-inflammatory drugs (NSAIDs) are the primary therapy options (1, 3, 4). NSAIDs are the initial choice for treating acute renal colic pain, which can decrease the frequency of hospital admissions but do not impact the passage of stones. However, they can cause renal dysfunction by decreasing renal blood flow and gastrointestinal discomforts and bleeding (5–7). On the other hand, opioids are useful but can cause drowsiness, respiratory depression, and dependency, hence alternatives are required (4).

Narcotics, which effectively manage pain via the central nervous system, are another medication useful for managing renal colic pain. However, due to their potential side effects, medical personnel should be in charge of administering them (8). A meta-analysis of randomized controlled trials showed that NSAIDs were equivalent to morphine alone in terms of reducing acute renal colic pain within the initial 30 min (5).

Gabapentin, an antiepileptic medication belonging to the class of drugs known as gabapentinoids, exhibits potential as an analgesic medication and is particularly recommended for managing neuropathic pain caused by diabetic neuropathy, restless legs syndrome, and other forms of central neuropathic pain (9–11). Recent research has demonstrated that gabapentin has analgesic properties for both acute pre- and postoperative somatic pain, reducing the need for opioids (12, 13). Many mechanisms, including calcium channel modulation, inhibition of excitatory neurotransmitter release, descending inhibition via enhancing serotonergic and noradrenergic systems, and reduction of temporal summation—the phenomenon where repeated stimuli cause progressively increased pain perception—are responsible for its effectiveness in managing somatic pain, especially in the context of postoperative and inflammatory pain (14–16).

Despite the administration of NSAIDs and morphine alone or in combination to patients with acute renal colic pain, referred to as ED, pain is frequently uncontrolled, necessitating frequent rescue doses of morphine.

The management of renal colic pain remains an issue in the emergency department. The addition of adjutant gabapentin to standard therapy may have beneficial impacts in lowering renal colic pain, but this effect has not been verified. This trial aims to evaluate the safety and efficacy of a 600 mg oral dose of gabapentin compared to a placebo in providing further pain relief for severe renal colic pain.

## 2 Material and methods

The investigation was conducted out as a double-blinded, randomized controlled clinical trial with two parallel treatment groups at Sina Hospital ED, which is affiliated with Tehran University of Medical Sciences in Tehran, Iran. This clinical trial was authorized by the Iranian Registry of Clinical Trials (registration code: IRCT20200322046833N2) and approved by the Research Ethics Committee of Tehran University of Medical Sciences (approval number: IR.TUMS.TIPS.REC.1399.154).

Prior to enrollment in the study, all eligible participants or their guardians provided written informed consent.

### 2.1 Study population

To be eligible to participate in the present study, all following precipitants, aged 18 to 85 years, who presented with renal colic pain on a visual analogue scale (VAS) (17) more than 6, which is confirmed by computed tomography (CT) scan or ultrasonography were screened. Patients having a VAS score (a numerical rating scale ranging from 0 (no pain) to 10 (worst pain imaginable)) of at least 6 (severe pain) were given parenteral ketorolac and morphine in accordance with our hospital's pain management strategy.

Exclusion criteria included the following: history of chronic opioid use, oral intake intolerance, active peptic ulcer, active COIVD-19 infection, history of renal failure (stage 4 and beyond in chronic kidney disease) or liver impairment (Child-Pugh grades B and C), pregnancy, lactation, recent trauma, use of any analgesia within 6 h of participation, taking Gabapentin or Pregabalin within the previous 7 days, chronic lung disease (e.g., pulmonary fibrosis, asthma, etc.), and concurrent participation in other studies were excluded.

### 2.2 Sample size calculation

Goodarzi et al.'s study (15) guided the sample size calculation, ensuring a power analysis to detect a clinically meaningful difference in pain reduction between the gabapentin and placebo groups. An effect size of 1 and standard deviation of 1.4 were considered, indicating a moderate difference in pain scores. At a significance level of 0.05 and a power of 80%, it was calculated that 66 patients would be enough to obtain the required statistical power. This computation, by reducing the possibility of Type II errors, ensures that the study has sufficient power to identify significant differences between the groups.

According to Goodarzi et al.'s study (18), assuming a confidence level of 0.05 and a margin of error of 80%, the minimum sample size for each group will be 33 patients.

### 2.3 Randomization

An online tool from https://www.sealedenvelope.com was used to execute out the block randomization process. A block size of four was selected in considering to guarantee a fair distribution of participants between the groups. The 66 individuals were distributed into 16 groups of 4 and one group of 2. In each block, a matched random sequence, such as AABB or ABAB, was generated. A non-affiliated researcher, who was not part of the trial, employed a random sequence to allocate patient to either the gabapentin or placebo group, so reducing selection bias and upholding the integrity of the study.

### 2.4 Blinding

The hard gelatin capsules containing gabapentin and the placebo were similar. The placebo capsules contained the same amount of powder.

The allocated groups were unknown to anyone (contain patients, physicians, research students, and statisticians) other than the principal investigator.

### 2.5 Intervention

The participants in the intervention and control groups were administered two capsule 300 mg (a total of 600 mg) of gabapentin (manufactured by Mehrdarou Company of Iran, under the trade name Neuropentin) or a placebo that was matched to the gabapentin, respectively, upon enrollment. Moreover, an intramuscular injection of 30 mg of ketorolac was administered to each patient at the same time with gabapentin (maximum 10 min apart).

Morphine sulphate was given intravenously as rescue therapy when the VAS was six or higher (0.05 mg/kg as the initial dose, then upgraded by 0.03 mg/kg) until the VAS was continuously decreased to less than 5.

### 2.6 Monitoring

The Visual Analog Scale (VAS), a commonly used pain intensity measure, was used to quantify pain severity. The VAS is a 10-cm horizontal line with “no pain” (0) and “worst imaginable pain” (10). We marked a point on the line for each post-intervention time period to indicate the severity of pain. The VAS score measured the centimeter distance from the left endpoint to the participant's mark, indicating pain severity. This approach measures a continuous range of pain intensity, enabling the precise measurement and comparison of gabapentin and placebo pain levels. The VAS assessment timepoints were at baseline, then every 5 min until their VAS score dropped to less than or equal to 7, then every 15 min until score 4 or lower.

Vital signs (including heart rate, blood pressure, respiration rate, and oxygen saturation) and probable side effects (including hypotension, bradypnea, nausea and vomiting, pruritus, a lowered state of consciousness, dizziness, and tiredness) were recorded during therapy at 15 min intervals of up to 120 min. If subjects experienced severe adverse reactions that necessitated awareness of the experimental medication, they were unblinded, which was not happened in this study.

### 2.7 Outcomes

The primary outcome of the study was the average pain severity measured using the VAS method at 40, 60, and 90 min.

The need for rescue morphine sulphate within the first 20 min of the study, the total amount of morphine sulphate used in milligrams (mg) and milligrams per kilograms (mg/kg) of the patient's weight, the average time taken to achieve a VAS score of less than 5, and a comparison of the two groups’ likely side effects were the secondary outcomes.

### 2.8 Statistical analysis

Potential confounders that could affect the study's results were taken into account in our analysis, including patient age, baseline pain levels, and hemodynamics statues.

Following the completion of data collection, statistical analysis was conducted using the STATA-17 statistical software program. The Kolmogorov-Smirnov test was used to examine the quantitative data for normal or non-normal distribution. If the data distribution followed a normal distribution, the findings were presented as the mean plus or minus the standard deviation (SD) and compared between the two groups using a *t*-test. If the data distribution deviated from normality, the findings were represented by the median [P25 P75], and their comparison between the two groups was conducted using the Mann-Whitney test. The qualitative data were presented as frequency and percentage, and Fisher's exact test was employed to compare them. A *p*-value below 0.05 was deemed statistically significant in this investigation.

## 3 Result

**Figure 1:**
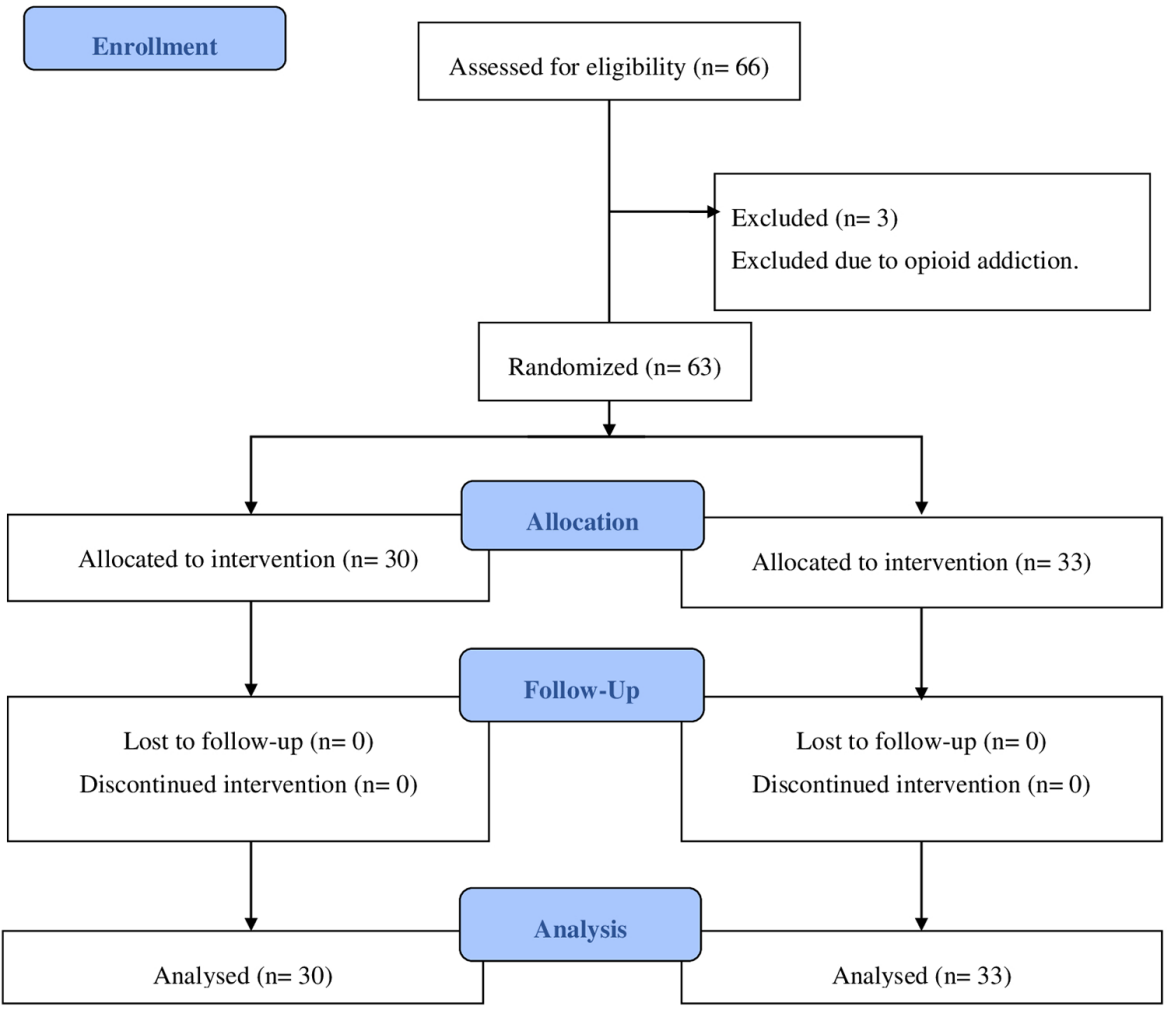
Consort diagram of study.

A total of 82 patients underwent screening from June 2021 to November 2021. Out of the total number, 66 patients met the requirements of the study and were selected at random to be divided into two groups: 33 patients received gabapentin and 33 patients received a placebo. This division occurred after the patients completed the informed consent form to participate in the trial. Three patients in the gabapentin group were excluded from this study due to their opioid's addiction (Figure 1).

Except for weight, the two groups’ baseline characteristics were comparable. The average weight of patients in the placebo group is significantly higher (*p*-value = 0.005). It should be noted that there was no significant difference between the two groups’ baseline vital signs or history of prior renal colic pain.

It is noteworthy that both groups’ median VAS scores at the start of the intervention were between 8 and 10, and there was no statistically significant difference between them in this regard (*p*-value = 0.80) (Table 1).

**Table 1:** Baseline demographic characteristics.

Variables	Gabapentin *N* = 30	Control *N* = 33	*p*-value
Age Median[P_25_P_75_]	38.5 [29.75 52.75]	38 [34.5 45]	0.70
Weight (kg) Mean ± SD	76.10 ± 12.77	85.76 ± 13.10	0.005
Gender Female/Male (%)	9/21 (30/70%)	4/29 (12.1/87.9%)	0.075
VAS at the baseline Median[P_25_P_75_]	10 [8 10]	10 [8 10]	0.80
Nausea Frequency (%)	16 (53.3%)	19 (57.6%)	0.466
History of Renal Colic Frequency (%)	16 (53.3%)	20 (60.6%)	0.372
SBP (mmHg) Median[P_25_P_75_]	130 [122.5 145.5]	129 [120 137.5]	0.37
RR (beat/minutes) Median[P_25_P_75_]	22 [20 26]	22 [20 23]	0.19
HR (beat/minutes) Median[P_25_P_75_]	78.5 [66.5 87]	79 [72.5 85]	0.5

IQR, interquartile; Kg, kilogram; SD, standard deviation; VAS, visual analogue scale; SBP, systolic blood pressure; RR, respiratory rate; HR, heart rate.

As described in Table 2, forty minutes after the intervention, patients in the gabapentin group had superior control over their pain intensity (VAS of less than five, 76.7% vs. 54.5% in the placebo group). However, the difference in VAS scores was not statistically significant (*p*-value = 0.057). After 60 and 90 min, respectively, the gabapentin group experienced a significant decrease in pain intensity (*p* = 0.03, 0.04 for VAS <5).

**Table 2:** Frequency of patients reported VAS of pain less than 5 at different time intervals.

VAS < 5	Gabapentin group *N* = 30	Control group *N* = 33	*p*-value
At 40 min Frequency (%)	23 (76.7%)	18 (54.5%)	0.057
At 60 min Frequency (%)	26 (86.7%)	21 (63.6%)	0.034
At 90 min Frequency (%)	29 (96.7%)	26 (78.8%)	0.037
Discharge in 90 min	22/30 (73.3)	16/33 (48.5)	0.07

VAS, visual analogue score.

The discharge rate in 90 min of enrollment in the gabapentin and placebo groups was 73.3% and 48.5%, respectively, which is statistically marginally not significant (*p*-value = 0.07).

Table 3 shows the VAS measurements for pain at different time points in between groups. Although it was not statistically significant in minutes 40 and 60 (*p*-value = 0.69 and 0.24, respectively), pain alleviation began faster in the gabapentin group. The gabapentin group showed a significant decrease in pain at minute 90 when compared to the control group (*p*-value = 0.02), where all patients were pain-free at the 120th minute.

**Table 3:** Visual analogue score (VAS) of pain changes during the time.

Time from participation	Gabapentin group *N* = 30	Control group *N* = 33	*p*-value
Minute 0 median (IQR)	10 [8, 10]	10 [8, 10]	0.96
Minute 40 median (IQR)	2 [3.5, 5]	4 [0, 6]	0.69
Minute 60 median (IQR)	1 [0, 3]	3 [0, 6]	0.24
Minute 90 median (IQR)	0.0 [0.00, 1]	1 [0, 5]	0.02
Minute 120 median (IQR)	0.0 [0.0,0.0]	0.0 [0.0, 3.5]	0.00
VAS difference from baseline at 40 min	−9 [−9,−7.75]	−9 [−10,−8]	0.513
VAS difference from baseline at 60 min	−9 [−9,−7.75]	−9 [−10,−7.5]	0.442
VAS difference from baseline at 90 min	−9 ± 0.53	−4.41 ± 1.73	0.012

IQR, interquartile.

The VAS difference at 90 min was statistically lower in the gabapentin group compared to controls (−9 ± 0.53 vs. −4.41 ± 1.73, *p*-value = 0.012), despite the fact that the VAS difference from baseline at 40 and 60 min was not statistically different (*p*-value = 0.513 and 0.442, respectively), which is presented at Table 3.

Table 4 demonstrates that the gabapentin group had considerably lower total morphine consumption and frequency of rescue morphine in the first 20 min of the study (*p* = 0.03).

**Table 4:** Morphine intake in each group of the study.

Variables	Gabapentin	Control	*p*-value
Need for morphine rescue in first 20 min of participation. Frequency (%)	6 (20%)	15 (45.5%)	0.03
The total dose of Morphine (mg) median (IQR)	4 [3, 5]	6.5 [4, 7.5]	0.003
The initial dose of morphine (mg) median (IQR)	0.05 [0.049, 0.0503]	0.05 [0.047, 0.053]	0.604
The Total Dose Of Morphine Per Weight (Mg/Kg) Median (IQR)	0.050 [0.049, 0.059]	0.076 [0.050, 0.082]	0.033

Mg, milligram; IQR, interquartile.

Despite that the initial weight-based morphine dose in both groups was statistically comparable (*p*-value = 0.604), the placebo group's total morphine consumption (measured in milligrams) was considerably higher (*p*-value = 0.003). Although the higher total morphine intake may be related to the higher baseline weight of its patients, total morphine intake in terms of mg/kg in the placebo group was still higher than that in the gabapentin group (*p*-value = 0.033). Above finding was presented in Table 4.

None of the two groups’ patients experienced serious adverse effects during the trial that required stopping their medication or implementing therapeutic controls to manage the issue.

## 4 Discussion

This study was a double-blind randomized clinical trial that aimed to examine the impact of gabapentin, when used alongside the conventional analgesic regimen of ketorolac and morphine, on patients experiencing acute renal colic pain. According to the study's findings, patients with acute renal colic who receive gabapentin in addition to a conventional analgesic regimen report a significant reduction in pain intensity within the first hour of treatment. Additionally, patients use less morphine overall and require rescue morphine less frequently. Furthermore, no serious side effects or complications were reported in any of the individuals under study.

This study provides crucial insights into the management of acute renal colic pain by highlighting the potential advantages of gabapentin as an addition to standard analgesic regimens such as ketorolac and morphine. Severe pain is a hallmark of renal colic, which frequently necessitates significant analgesic intervention. Within the first hour of the study's enrollment, gabapentin dramatically lowered pain intensity and reduced the requirement for morphine; this suggests that gabapentin may play a key role in improving pain management measures for these individuals. These results have clinical value since they demonstrate that gabapentin may successfully reduce pain, which can lead to improved patient outcomes in emergency settings. For patients with conditions like renal colic, where the pain can be debilitating, pain management is an essential part of their therapy. Gabapentin may provide a safer alternative or complementary strategy to pain management by lowering reliance on opioids, which have been linked to side effects such as respiratory depression and possible dependency.

There are a variety of different mechanisms that contribute to the efficacy of gabapentin in the management of somatic pain in postoperative and inflammatory conditions. These approaches are accountable for the effectiveness. These mechanisms include the modulation of calcium channels, the inhibition of excitatory neurotransmitter release, the descending inhibition that occurs through the enhancement of serotonergic and noradrenergic systems, and the reduction of temporal summation, which is the phenomenon in which repeated stimuli cause progressively increased pain perception (14–16).

In only one relevant study at the time of this trial, gabapentin was shown to considerably lower pain levels and the need for opioids in patients with renal colic when assessed in a comparable setting by Goodarzi et al. (18). In contrast to the current trial, where over 85% of patients in the gabapentin group had mild pain (VAS < 5) after an hour, the study found that the majority of patients continued to feel moderate pain (VAS > 5). Given that the current trial utilized ketorolac, a strong NSAID, which may have led to more effective pain management, whereas Goodarzi et al. used pethidine without NSAIDs, the gap may be explained by changes in the baseline analgesic regimes.

Neuropathic pain and reflex sympathetic dystrophy are the only conditions in which gabapentin is primarily used to treat pain (19). Due to their ability to lower perioperative hyperalgesia, gabapentinoids have been included in postoperative pain therapy over the last decade (20).

Numerous research investigations have demonstrated that gabapentin exhibits a noteworthy impact on pain severity and the amount of opioids used post-operatively, in addition to working in concert with other painkillers (21–23). These effects could be resulting from a variety of ways, although the specific mechanisms remain unknown.

Unlike opioids, gabapentin acts by binding to the alpha-2-delta subunits of voltage-gated calcium channels, lowering spinal cord irritation and the release of neurotransmitters such substance P, noradrenaline, and glutamate in the pain pathways (23).

According to a 2015 systematic review, the most effective approach for minimizing postoperative pain after various types of surgeries is to administer gabapentin before to the procedure (24), but its effectiveness as adjutant medicine for lowering postoperative pain was mainly demonstrated to be unreliable by a thorough meta-analysis and review of all trials on this topic and the evidence is not sufficient yet (25–28).

By interfering with calcium channels, GABA analogs have the potential to effectively treat both peripheral and central pain as well as renal colic pain. They can also ease pain and facilitate the passage of kidney stones by widening the ureteral smooth muscle vessels (29).

These mechanisms suggest that gabapentin, which was also identified in our investigation, deserves to be able to effectively reduce acute renal colic pain. Thus, patients with renal colic pain may expect a faster reduction in pain intensity and a decrease in morphine intake when gabapentin is added to the conventional analgesic regimen.

A limitation of this study was the limited sample size of the study, which may have resulted in imprecise findings. While blinding all trial participants helped to mitigate this issue, it appears that a multicenter investigation with a bigger sample size is required. Although the VAS is a common grading system for determining pain severity, using it in conjunction with functional (30) and behavioral (31) indications can increase accuracy.

Overall, this study adds to the broadening body of data supporting the use of gabapentin as an adjuvant in conventional analgesic regimens, especially in acute pain conditions where opioid-sparing methods are becoming increasingly important. According to the research, gabapentin may improve pain management and lessen the demand for opioids, providing a potentially safer and more efficient method of treating acute renal colic pain.

## 5 Conclusion

This study shows that adding gabapentin to a ketorolac-based regimen significantly reduces acute renal colic pain severity and opioid analgesic use. Gabapentin may help manage acute renal colic pain, reducing narcotic consumption and improving emergency patient outcomes. Further study is needed to validate these findings and expand this approach's usefulness.

## Data Availability

Publicly available datasets were analyzed in this study. This data can be found here.
